# Efficacy of vitamin D3-fortified-yogurt drink on anthropometric, metabolic, inflammatory and oxidative stress biomarkers according to vitamin D receptor gene polymorphisms in type 2 diabetic patients: a study protocol for a randomized controlled clinical trial

**DOI:** 10.1186/1472-6823-11-12

**Published:** 2011-06-22

**Authors:** Sakineh Shab-Bidar, Tirang R Neyestani, Abolghassem Djazayery

**Affiliations:** 1 Department of Nutrition and Biochemistry, School of Public Health, Tehran University of Medical Sciences (TUMS), Tehran, Iran; 2 Laboratory of Nutrition Research, National Research Institute and Faculty of Nutrition and Food Technology; Shahid Beheshti University of Medical Sciences (SBUM), Tehran, Iran

**Keywords:** vitamin D, vitamin D receptor, polymorphism, type 2 diabetes, study protocol

## Abstract

**Background:**

Development of type 2 diabetes mellitus (T2DM) is determined by the interactions of genetic and environmental factors. This study was designed to evaluate the possible role of VDR single nucleotide polymorphisms (SNPs) on different aspects of diabetic host response (anthropometric, metabolic, oxidative stress and inflammatory) to daily intake of vitamin D through fortified yogurt drink for 12 weeks.

**Methods/Design:**

This study comprises two parts: (i) a case-control study; and (ii) an intervention trial. In the first part, VDR polymorphisms *(Taq1*, *FokI*, *Apa1*, *Bsm1*, and *Cdx2) *are determined in 350 T2DM patients and 350 non-diabetic subjects. In the second part, the possible effects of daily intake of two servings of vitamin D3-fortified yogurt drink (FYD; 500 IU vitamin D/250 mL) on some selected metabolic (including insulin resistance), inflammatory and oxidative stress biomarkers in 135 T2DM patients are assessed. To relate the resulted changes in the biomarkers to vitamin D replenishment, another group of diabetic patients (n = 45) are also included in the study who receive 2 servings of plain yogurt drink (PYD) a day. The primary outcome is serum level of 25(OH) D, which it is expected to be elevated only in FYD group. Secondary outcomes include improvements in glycemic, metabolic, inflammatory and oxidative stress biomarkers in FYD group compared to PYD group. Three VDR *FokI *polymorphisms are determined only in FYD group followed by comparison of changes in the biomarkers among these genotypic variants.

**Discussion:**

The present study, at least in part, elucidates the discrepancies in the results of different vitamin D-diabetes studies pertaining to the genetic variations of the population. If VDR polymorphisms are found to influence the response to our intervention, then knowing distribution of VDR polymorphisms in both diabetic and non-diabetic populations can give a picture of the proportion of the community in whom up to 1000 IU/d vitamin D may not be effective enough to improve insulin resistance and related morbidities. Therefore, they should ideally receive further nutritional support according to their genotype.

**Trial Registration:**

ClinicalTrials.gov: NCT01236846

## Background

Type 2 diabetes mellitus (T2DM) is a global epidemic costly condition [1]. Individuals with diabetes sustain reduction in quality of life, and high prevalence of mortality [2].

Development of T2DM is determined by the interactions of genetic and environmental factors [3,4]. Most, if not all, genetic influences have a polymorphic nature [5,6]. A number of loci have been studied so far to explain the genetic susceptibility to T2DM. An Example is genes associated with glucokinase, hepatocyte nuclear factors, insulin promoter factors, possibly the insulin and insulin receptor genes and mitochondrial genes [7]. Among interesting, and less studied, diabetes-related genes are those associated with vitamin D metabolism.

Apart from its calcemic effects, vitamin D is now known to have many non-calcemic functions through which it may have some role in several human pathologies including some forms of cancers [8], autoimmune disorders [9], obesity [10], metabolic syndrome [11] and both types 1 and 2 diabetes [12,13], among the others. Animal studies have shown impaired insulin secretion [14] and action [15,16] in vitamin D deficiency. Though vitamin D replenishment of type 2 diabetics has been shown to improve inflammatory biomarkers and glycemic control [17-23], some studies failed to demonstrate these beneficial effects [24,25]. The reasons for these discrepancies in results could be numerous including sample size, duration and mode of intervention (supplementation, injection, fortification, or...) and dosages used, baseline values of 25(OH)D and also of the other variables (glucose, lipids and so on), method of vitamin D assessment (RIA, CPBA, CL or HPLC) and finally genetic makeup of the study population.

The active form of vitamin D acts through a specific vitamin D receptor (VDR) [26]. Allelic differences in the VDR gene may contribute to the genetic predisposition to certain diseases [10]. Research findings on associations among VDR polymorphisms, vitamin D stats, T2DM and glycemic status have not been unanimous; some confirmed such relations [18] and some did not [25]. Nevertheless, as VDR has been recently found on pancreatic beta cells [24], it is plausible that genetic variants of the VDR gene may contribute to the development of T2DM. There is a mere paucity of controlled clinical trials on vitamin D and T2DM. On the other hand, the findings of a few trials have been inconsistent. Those studies mostly used either vitamin D injection (monthly or weekly high dose injection) [27,28] or supplements [16,29,30] (usually with dosages less than the amount believed to be sufficient [31]). In the present study we are going to use yogurt drink, which is an Iranian drink (Persian name: Doogh), for vitamin D fortification. Using fortified foodstuffs have the advantage of lower cost and higher compliance [32]. Considering the common nature of T2DM with obesity-induced insulin resistance and metabolic syndrome [33], two conditions with growing occurrence which may lead to T2DM [34], using vitamin D3-fortified yogurt drink, if proved to be effective in this study, can be encouraged as a preventive tool. This idea must be substantiated by further cohort studies.

This study was designed to evaluate the possible role of VDR single nucleotide polymorphisms (SNPs) on different aspects of diabetic host response (anthropometric, metabolic, oxidative stress and inflammatory) to daily intake of vitamin D through fortified yogurt drink for 12 weeks.

## Specific Aims

This study comprises two parts. The first part aims to quantify the magnitude of association between any identified variants of the VDR gene and T2DM. A cross-sectional study is employed to compare distribution of each genotype of VDR polymorphisms in 350 type 2 diabetic adults and 350 non-diabetic controls. The second part is outlined to compare within- and between-polymorphic group variations of host response to daily intake of vitamin-D3-fortified yogurt drink in terms of anthropometric, metabolic, inflammatory and oxidative stress markers.

### Part 1

#### Primary Objectives

To determine distribution of VDR gene polymorphism genotypes (*Taq1*, *FokI*, *apa1*, *Bsm1*, *Cdx1*) in T2DM patients and non-diabetic volunteers and to compare between these two groups

#### Secondary Objectives

To determine distribution of VDR gene polymorphism haplotypes (*Taq1, FokI, Apa1, Bsm1, Cdx2*) in T2DM patients and non-diabetic controls and to compare between these two groups

### Hypothesis

(i) There is a difference in prevalence of vitamin D receptor gene polymorphisms between diabetics and non-diabetic controls

(ii) There is a difference in prevalence of vitamin D receptor gene polymorphism haplotypes between diabetics and non-diabetic controls

### Part 2 study

#### Primary Objectives

To compare within- and between-polymorphic group variations in circulating 25(OH)D in diabetic subjects and also between diabetics and non-diabetic subjects.

#### Secondary objectives

(i) To compare within- and between-polymorphic group variations in anthropometric, metabolic, inflammatory and oxidative stress biomarkers

(ii) To conduct an exploratory analysis of changes in mean of anthropometric, metabolic, inflammatory and oxidative stress biomarkers in FYD group among sub-groups defined by genotypes of each VDR polymorphisms.

### Hypothesis

(i) Daily consumption of vitamin D3-fortified-yogurt drink improves serum 25(OH)D in type 2 diabetic patients.

(ii) Daily consumption of vitamin D3-fortified-yogurt drink improves biomarkers of anthropometric, metabolic, inflammatory and oxidative stress in type 2 diabetes patients.

(iii) Response to daily consumption of vitamin-D3-fortified yogurt drink differ between VDR polymorphic variant groups

## Methods/Design

### Part 1

#### Participants

A case-control study is designed to compare VDR gene polymorphism distribution between T2DM and age-, sex- and body mass index (BMI)-matched non-diabetic subjects. T2DM patients are recruited from two centers; Iranian Diabetes Society and Gabric Diabetes Society, both located in Tehran. Diabetic subjects are recruited either by attending the classes held at the two centers and verbal invitation or through phone call. Non-diabetic controls are enrolled from school teachers, workers of Tehran or Shahid Beheshti Universities and Pegah Dairy Companies, all located in Tehran.

Full information on the study design and objectives of the study is given to all participants before they sign an informed written consent.

#### Sample Size

For observational study the sample size needed to obtain 90% power at a 5% significance level with an equal number of case and controls for the chi-square test on genotypes, is 350 at each group of cases and controls. The sample size is calculated using the software Quanto: http://hydra.usc.edu/gxe/.

#### Blood sampling

Fasting venous blood samples are collected into tubes either with or without anticoagulant (ethylene diamine tetra-acetate, EDTA). The anticoagulated tube is used to extract genomic DNA while the sera from clot samples are analyzed for glucose and lipids. Further information about blood sampling and genotyping are in part 2 study in detail.

#### Variables

Information on demographic, medical history, physical activity, duration of direct sun exposure and smoking habits are gathered using questionnaires and face-to-face interview. Anthropometric measures are taken in the same meeting. Table 1 shows the study variables.

**Table 1:** Study measures

Variable	Methods
***General data***	

**Demographic data**	Questionnaire

**Medical history**	Questionnaire

**Sun exposure**	Questionnaire

***Anthropometric and clinical data***	

**Height, weight, waist and hip circumference**	Stadiometer, digital scale (Seca 840, Germany)

**Blood pressure**	A digital sphygmomanometer (BC08, Beurer, Germany).

***Blood data***	

**Fasting plasma glucose, lipid profile**	Pars Azmoon, Tehran, Iran

**Complete blood cell count**	Mythic, Orphe'e, France

**VDR gene polymorphisms**	PCR-RFLP, Corbett Rotor-Gene 6000 instrument (Corbett Life Science)

#### Value of results

This is the first study on the distribution of VDR SNPs among type 2 diabetics and their possible relations to the particular phenotypes in Iran. These data assist both to the better understanding of vitamin D-T2DM inter-relationships and to the preventive programs.

### Part 2

**Figure 1:**
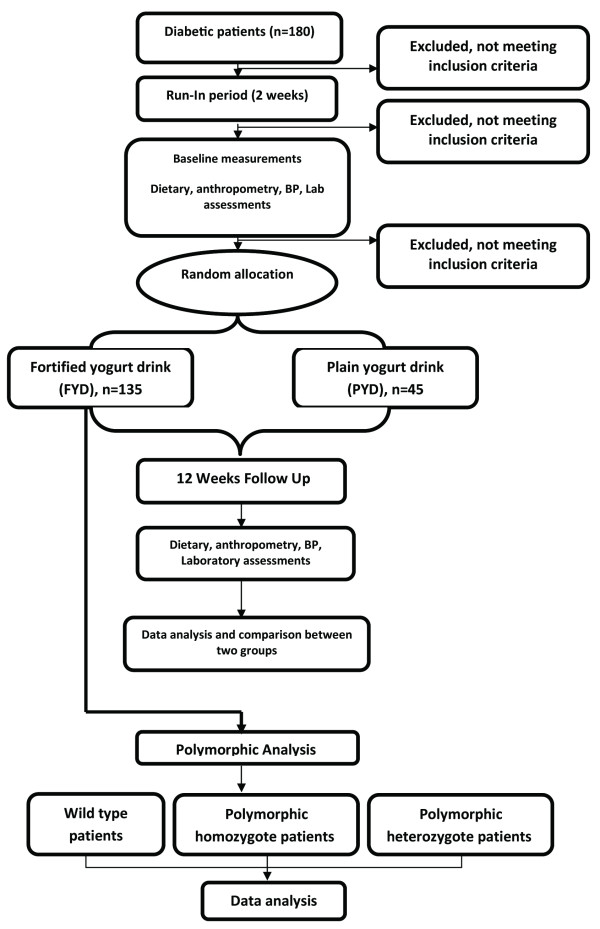
**A summary of the interventional study**.

This is a 3-month randomized controlled clinical trial (RCT) in which T2DM patients aged 30-60 years are enrolled. The study design is presented in Figure 1.

#### Setting

The possible effects of daily intake of two servings of vitamin D3-fortified yogurt drink on some selected metabolic (including insulin resistance), inflammatory and oxidative stress biomarkers in T2DM patients (n = 135) are assessed. To relate the resulted changes in the biomarkers to vitamin D replenishment, another smaller group of diabetic patients (n = 45) are also included in the study who receive 2 servings of plain yogurt drink a day. To minimize endogenous vitamin D synthesis, the entire intervention is confined to cold seasons (fall and winter) of the year. The RCT is conducted collaboratively by National Nutrition and Food Technology Research Institute (NNFTRI) and Tehran University of Medical Sciences (TUMS) in Tehran, the capital of Iran.

#### Participants

T2DM patients are recruited from Iranian Diabetes Society or Gabric Diabetes Society as described in the first part of the study. Volunteers are invited to attend Laboratory of Nutrition Research at NNFTRI while they are fasting. Data gathering, bleeding and urine sampling all are done during 07.30 and 10.00 am.

#### Inclusion criteria

1. Having type 2 diabetes

2. Willingness to participate

3. BMI between 25 to 35 kg/m^2^

4. Age: 30-60 years old

5. No use of any vitamin, dietary, herbal or omega-3 supplements since at least 3 months to and during the intervention period

#### Exclusion criteria

1. History of cardiovascular, gastrointestinal, renal, and other endocrinological diseases

2. Subjects receiving vitamin D, omega-3 or other types of dietary supplements

3. Pregnancy or lactation

4. Treatment with insulin

5. Treatment for weight reduction

6. Treatment for reducing serum cholesterol

#### Sample Size

Based on data on serum 25(OH)D changes from other studies [35], to achieve 1 standard deviation difference in circulating 25(OH)D with two-sided alpha of 0.05 and a beta of 0.2 after a 12-week intervention period, the sample size was calculated 40 individuals per treatment group. Considering 3 polymorphic variant groups and one control group which receives plain yogurt drink, 160 subjects are needed. Allowing for 10% attrition over 12 weeks of intervention, a total of 180 subjects are required.

#### Run-in

On the first visit and following completion of the questionnaires, all participants are given some general information on diabetic diet, based on American Dietetic Association (ADA) guidelines [36], with special focus on fruits, vegetables and dairy intakes. Patients are instructed not to change their usual physical activity and to include 2-3 exchanges of fruits, 2 exchanges of vegetables and 2-3 exchanges of low-fat milk exchanges in their daily diet for the next 2 weeks (run in period).

#### Randomization/Blinding

After run-in period, participants are assigned randomly to one of the treatment groups of either fortified yogurt drink (FYD, n_1 _= 135) or plain yogurt drink (PYD, n_2 _= 45) using permuted blocks of random numbers allocation method. Letter codes A and B are used to identify two groups, respectively. Yogurt drinks are identical in color, size, taste and packaging. Therefore, participants are not aware of their group, neither are all the interviewers and laboratory staff. However, the main investigators could not be technically blind to the identity of the letter codes.

The randomization and labeling process are audited halfway through the study by an observer to find if it is robust, accurate and able to maintain blinding of participants.

#### Intervention

Yogurt drinks are either plain (PYD; containing 170 mg calcium and no vitamin D/250 mL) or vitamin D_3_-fortified (FYD; containing 170 mg calcium and 500 IU vitamin D_3_/250 mL). Participants are instructed to consume a bottle of yogurt drink with both lunch and dinner, i.e. 500 mL/d equaling 1000 IU vitamin D_3 _a day in FYD group. The intervention period is 12 weeks. Consumption of 1000 IU/d vitamin D is believed to be safe and effective to increase circulating 25(OH)D [13,37,38]. Participants are given yogurt drinks in 30-bottle packs, which are enough for 2 weeks. All subjects are visited biweekly to both assess their compliance and provide them with yogurt drinks for the next two weeks. Blood pressure, anthropometric, dietary, body fat and laboratory evaluations are preformed both before and after intervention. Data comparison are made between PYD and FYD groups and also, in FYD group, among VDR polymorphic groups.

#### Compliance

All participants are given a pamphlet on "instructions to use yogurt drinks" together with a "yogurt drink consumption table" comprised of 28 empty boxes for each week. Subjects are instructed to mount the table in an exposed place (preferably on refrigerator) and to tick each box after consumption a yogurt drink with each meal. Moreover, they are asked to bring the empty bottles back on their next visit. Compliance is evaluated by checking the consumption tables, counting the empty bottles, direct enquiry both on the biweekly visits and weekly follow-up phone calls.

#### Quality control of product

Composition (including vitamin D_3_) of the yogurt drinks are measured right after production, in the middle and end of the storage, to ensure the stability of the components, especially vitamin D, in the product. The measurements are done by Maad Laboratory of Foods, Drinks and Cosmetics, accredited by the Deputy of Food and Drug of the Iranian Ministry of Health.

#### Outcome Measurements

##### Dietary assessment

A semi-quantitative Food Frequency Questionnaire (sqFFQ) and 24-h dietary recall for 3 days (including a weekend day) are used for assessment of dietary intake.

##### Anthropometry and Blood Pressure

Body weight is measured with light clothes without shoes using a digital scale (Seca 808, Germany) to the nearest of 0.1 kg. Height is measured using a stadiometer (Seca, Germany) to the nearest of 0.1 cm. BMI is calculated by dividing weight (kg) per height (m) square. Waist circumference (WC) is measured in the midpoint of the lowest rib and iliac crest in the end of expiration using a measuring tape to the nearest of 0.1 cm

To measure blood pressure (BP), subjects are allowed to be in sitting position at least 10 minutes. BP is measured twice by a digital sphygmomanometer (BC08, Beurer, Germany). The average of the two measurements is considered as subject's BP.

##### Body Composition Analysis

The percentage of body fat is evaluated using bioelectrical impedance analysis (Quadscan 4000 system, Bodystat, UK).

### Laboratory investigations

All laboratory analyses are performed at the Laboratory of Nutrition Research, NNFTRI.

#### Blood sampling and handling

Twenty mL of fasting venous blood taken from all participants by phlebotomy is divided in three tubes: (i) a micotube with an anticoagulant, EDTA; (ii) a heparinized 15-mL sterile capped plastic tube; and (iii) a clean glass test tube without anticoagulant. Of about 500 μL EDTA-blood sample in the microtube, 9 μL is used for CBC. Heparinized blood sample is used for PBMC separation and culture while the clot blood samples are kept for ~30 at RT followed by centrifugation at 1500 *g *at RT. Sera thus recovered are transferred to clean micro-tubes in aliquots. One aliquot is used to determine fasting serum glucose (FSG) and lipid profile in the same day of bleeding while the other tubes are kept at -80°C until the day of analysis.

#### Hemoglobin and hematocrit

Hemoglobin and hematocrit are measured as parts of complete blood cell count (CBC) test in the same day of blood sampling by a cell-counter system (Mythic, Orphee, France).

### Blood chemistry

Fasting serum glucose (FSG), lipid profile including triglycerides (TG), total cholesterol, low-density lipoprotein cholesterol (LDL-C), and high-density lipoprotein cholesterol (HDL-C), alanine aminotransferase (ALT), aspartate aminotransferase (AST) and uric acid are determined using enzymatic methods. Serum total protein, albumin, calcium, phosphorous and magnesium are measured by colorimetric assays. All these tests are done by commercial kits (all from Pars Azmoon, Iran) using an auto-analyzer system (Selectra E, Vitalab, the Netherlands).

Glycated hemoglobin (HbA1c) is determined using colorometric method after an initial chromatographic separation (BioSystems, Spain).

Serum insulin levels are measured by immunoradiometric assay (IRMA) using a commercial kit (Biosource, Belgium) and a γ-counter system (Gamma I, Genesys, USA).

Insulin resistance is evaluated by Quantitative Insulin Check Index (QUICKI) calculated as [39]:

QUICKI: 1/[log (insulin) (μU/mL) + log (glucose) (mg/dL)]

Serum 25(OH)D concentrations are measured by HPLC method as described elsewhere [40]. Serum intact parathyroid hormone (iPTH) (DRG, Germany) and osteocalcin (Biousource, Belgium) concentrations are determined using enzyme immunoassay (EIA).

In this study oxidative stress is evaluated by determination of serum glutathione, glutathione peroxidase, glutathione reductase, superoxide dismutase (all from Bender Medsystems, Austria), total antioxidant capacity (TAC) using colorometic method with ABTS reagent and bovine serum albumin as standard, as reported earlier [41] and malondialdehyde (MDA), as described originally [42] with some modifications [43].

Inflammatory status is evaluated using determination of highly sensitive C-reactive protein (hsCRP) (immunoturbidometric assay, Pars Azmoon, Iran), serum amyloid A (SAA), endothelin-1 (both from IBL, Germany), E-selectin and matrix metalloproteinase (MMP)-9 (both from e-Bioscience, Austeria).

### Urinary tests

Urinary sodium and potassium are measured by flame photometry (Corning 480; Corning Ltd, Halstead, Essex, UK). Urinary albumin and Creatinine are determined by immunoturbidometric and colorometic methods, respectively (both from Pars Azmoon, Iran) with the aid of an auto-analyzer (Selectra E, Vitalab, the Netherlands).

#### PBMCs culture

Peripheral blood mononuclear cells (PBMC) were separated using Ficoll-hypaque gradient density centrifugation and cultured as described elsewhere [44] with minor modifications. In this study, ~10^6 ^cells are transferred to a culture well in a 6-well cell culture plate (Greiner bio-one, Germany), each well containing 1% phytohemmaglutinin (PHA) (Gibco), 100 ng/mL lipopolysaccharide (LPS) (Gibco), 100 U/mL penicillin G and 10 ng/mL streptomycin (both from Sigma-Aldrich) in a total volume of 2 mL RPMI 1640. PBMC culture is performed in triplicate.

#### Cytokine assay

Concentrations of interleukin (IL)-2, IL-4, IL-6, IL-10 and tumor necrosis factor (TNF)-α (all from Bender Medsystems, Austria) are determined in PBMCs culture supernatant. All EIA tests are performed with the aid of a microplate reader (Statfax 3200, Awareness, USA).

### DNA extraction

Whole blood collected in a microtube containing 20 μL 7% EDTA are processed using Genet Bio DNA Isolation Kit (PrimePrepth, South Korea) following the procedures detailed in the kit to extract genomic DNA. Stock DNA is stored at -20 in a linked anonymized format, with codes held by principal investigators.

### DNA genotyping

The VDR genotypes at *BsmI, TaqI, ApaI, FokI *and *Cdx-2 *SNP sites are determined using polymerase chain reaction (PCR)-restriction fragment length polymorphism (RFLP) analysis as described below. We use a gradient palm-cycler (Corbett Research, Australia) for PCR amplification. The purified primers (Fermentase) and Premixes (Cinnagen) are purchased. Specific primers are selected and validated for SNPs (table 2) [45-47]. We use BLAST search (http://www.ncbi.nlm.nih.gov/BLAST) to check the specificities and homology percent of primers for the VDR sequence. All primers show 99% homology to the sequence in specific region of the VDR gene.

**Table 2:** VDR polymorphisms and PCR-RFLP information

SNP	Localization	Base change	Nomenclature	Forward primer/reverse primer	Amplicone	Restriction site	Digestion fragments	Genotype	Restriction enzyme	Reference
***FokI***	Exon 2	C/T	FF Ff ff	5'-AgC Tgg CCC Tgg CAC TgA CTC TgC TCT-3' 5'-ATg gAA ACA CCT TgC TTC TTC TCC CTC-3'	265	CC CT TT	207-60	mut ht wt	FokI	[45]

***BsmI***	Intron 8	G/A	BB Bb bb	5'-ggC AAC CTg AAg ggA gAC gTA-3' 5'-CTC TTT ggA CCT CAT CAC CgA C-3'	461	AA GA GG	258-253	mut ht wt	Mva 1269I	[46]

***TaqI***	Exon 9	T/C	TT Tt tt	5'-CAg AgC ATg gAC Agg gAg CAA g-3' 5'-gCA ACT CCT CAT ggC TgA ggT CTC A-3'	740	TT TC CC	495-245-290-205	wt ht mut	TaqI	[45]

***ApaI***	Intron 8	G/T	AA Aa aa	5'-CAg AgC ATg gAC Agg gAg CAA g-3' 5'-gCA ACT CCT CAT ggC TgA ggT CTC A-3'	740	TT GT GG	515-225	mut ht wt	Bsp120I	[45]

***Cdx-2***	Intron 1e	G/A	CC Cc cc	5'-CAg CAT gCC TgT CCT CAg C-3' 5'-CCA gTA CTg CCA gCT CCC-3'	250	AA GA GG		mut ht wt	Bpu10I	[47]

### Data management

For the better quality and accuracy of data, real time data entry is ensured. We use manual checking of frequencies during data entry. For primary analysis, alphabetical codes for groups are used still blinded to the real group identity.

### Statistical analyses

Data are expressed as mean ± SD. Normality of data distribution is evaluated using Kolmogrov-Smirnov. Comparison of data between two groups is done by either student *t *test (normal distribution) or Mann-Whitney U (not normal distribution).

Genotype distribution of the 5 VDR SNPs in the study population is evaluated for Hardy-Weinberg equilibrium by Chi square test. Odds ratios (OR) are given with 95% confidence intervals (CI). Haplotypes frequencies and distributions are estimated with a program based on EH (estimating haplotypes)[48]. Haplotypes are compiled as combinations of the 3 SNPs (*BsmI*, *TaqI*, *ApaI*).

The differences in distribution of the biomarkers among polymorphic variant groups are compared by analysis of variance (ANOVA) and, in the case of significant difference, Tukey's post hoc analysis. A p value < 0.05 is considered significant. Correlation between two sets of data is evaluated using either Pearson (normal distribution) or Spearman (not normal distribution) equations. Multiple linear regression modeling is used to identify the contributions of each independent variable to the variation of response to vitamin D supplementation.

### Data Validation

Data cleaning takes place by a series of logical checks on the electronic data. Discrepant records are checked with the source documents and the database is amended, if necessary.

### Ethical issues

The study protocol has been approved by the NNFTRI (No. 035360), Shahid Beheshti University of Medical Science, and by Tehran University of Medical Sciences (TUMS) (No.10533) Ethical committees.

## Discussion

Increasing evidences suggest a pivotal role for vitamin D in insulin action [15,16] and T2DM [12,13]. However, the results of vitamin D supplementation in diabetics have not been consistent [20,25]. The reasons for this inconsistency could be the vitamin D dosage and route of administration (injection [27,28] vs. oral [30]), age of the study population (middle aged [49] vs. elderly [50]) and probably genetic variations. The role of VDR polymorphism in response to vitamin D interventions has been reported in other clinical settings [51,52]. The contribution of VDR polymorphism in susceptibility to T2DM has also been shown in some studies recently [18]. However, this is the first clinical trial on the role of VDR polymorphism in response to vitamin D replenishment on different aspects of diabetic pathology, including anthropometric, metabolic, oxidative stress, inflammatory and immunity. This study pursues three objectives: Firstly, to tackle relationship between VDR genotypes and T2DM for the first time in Iranian population; secondly, to elucidate the potential mechanisms and cross-talk among pathways, by which vitamin D exerts its effects on diabetes, insulin resistance and cardiovascular disease by evaluating an extensive panel of biomarkers (anthropometric, metabolic, inflammatory and oxidative stress); and finally to identify vitamin D responsive vs. less or (maybe) non-responsive VDR genetic variants in subjects with T2DM for further proper nutritional care.

The following points can be considered as the strengths of this study: i) the intervention is conducted during cold seasons, when dermal synthesis, especially at Tehran latitude, is minimal[53]; ii) various degrees of vitamin D deficiency have been shown to be prevalent in Iranian general population at almost all ages [54] as well as diabetics [21]. Because of lower basal 25(OH)D levels, the response to vitamin D replenishment is expected to be more prominent; iii) yogurt drink, a popular Iranian drink, is used to fortify with vitamin D for the first time. The results of this study will show the bioavailability of vitamin D in this drink. Moreover, apart from the added vitamin D, yogurt drink protein and calcium content makes it a very good choice and a substitute for sugar-free colas and even juices in diabetic, as well as a variety of non-diabetic, diets; iv) the dosage of vitamin D [55] and duration of intervention [56] seem to be proper.

The present study, at least in part, elucidates the discrepancies in the results of different vitamin D-diabetes studies pertaining to the genetic variations of the population. On the other hand, the importance of the upcoming results lies on the fact that both metabolic syndrome and obesity-induced insulin resistance, two conditions with increasing global occurrences which predispose T2DM development [33], have similar pathologic bases [34]. If VDR SNPs are found to influence the response to our intervention, then knowing distribution of VDR polymorphisms in both diabetic and non-diabetic populations can give a picture of the proportion of the community in whom up to 1000 IU/d vitamin D may not be effective enough to improve insulin resistance and related morbidities. Therefore, they should ideally receive further nutritional support according to their genotype.

## Abbreviations

BLAST: basic local alignment search tool; BP: blood pressure; BMI: body mass index; CVD: cardiovascular disease; CL: chemiluminesence; CPBA: competitive protein binding assay; DNA: deoxyribonucleic acid; EIA: enzyme linked immune assay; EDTA: ethylene diamine tetra-acetate; FSG: fasting serum glucose; FYD: fortified yogurt drink; HDL-C: high density cholesterol; ht: heterozygote mutated; HPLC: high-performance liquid chromatography; hs-CRP: highly sensitive C-reactive protein; iPTH: intact parathyroid hormone; IFN-gamma: Interferon-gamma; IL-4/IL-10/IL-6/IL-2: Interleukin 4/Interleukin 10/Interleukin-6/Interleukin-2; LPS: lipopolysaccharide; LDL-C: low density lipoprotein cholesterol; MDA: malondialdeyde; mut: homozygote mutated; NNFTRI: national nutrition and food technology research institute; PHA: phytohemmaglutinin; PYD: plain yogurt drink; PCR: polymerase chain reaction; RIA: radioimmunoassay; RCT: randomized clinical trial; RFLP: restriction fragment length polymorphism; sqFFQ: semi-quantitative Food Frequency Questionnaire; SNP: single nucleotide polymorphism; TUMS: Tehran University of medical sciences; TG: triglycerides; TNF: tumor necrosis factor; T2DM: type 2 diabetes mellitus; WC: waist circumference; Wt: homozygote wild type

## Competing interests

The authors declare that they have no competing interests.

## Authors' contributions

TRN designed the initial idea of this work, which was further developed by SSh and ADj. Now, this is a common project between NNFTRI and TUMS, the most part of which is being done as Ph.D. research of SSh under the supervision of both TRN and ADj. The manuscript has been read and approved by all authors.

## Pre-publication history

The pre-publication history for this paper can be accessed here:

http://www.biomedcentral.com/1472-6823/11/12/prepub
